# Coptidis Rhizoma extract mitigates sleep deprivation-induced cognitive impairment and neurodegeneration: Insights into hormonal, glymphatic, and molecular mechanisms

**DOI:** 10.1016/j.jtcme.2025.12.004

**Published:** 2026-01-10

**Authors:** Chih-Yuan Yang, Yea-Hwey Wang, Kuo-Tong Liou, Shuo-En Hsu, Cher-Chia Chang, Yen-Yu Chen, Terry B.J. Kuo, Hung-Tse Huang, I-Wen Lo, Chia-Ching Liaw, Yuh-Chiang Shen, Yi-Chang Su

**Affiliations:** a National Research Institute of Chinese Medicine, Taipei City, 112026, Taiwan; b Institute of Brain Science, National Yang Ming Chiao Tung University, Taipei City, 112304, Taiwan; c National Taipei University of Nursing and Health Science, Taipei City, 112303, Taiwan; d Department of Medicine, Mackay Medical College, New Taipei City, 252005, Taiwan; e Department of Chinese Medicine, Tri-Service General Hospital, National Defense Medical Center, Taipei City, 114202, Taiwan; f Department of Life Sciences, National Chung Hsing University, Taichung, 402202, Taiwan; g Institute of Pharmacology, School of Medicine, National Yang Ming Chiao Tung University, Taipei City, 112304, Taiwan; h Department of Psychiatry, Shin Kong Wu Ho-Su Memorial Hospital, Taipei City, 111045, Taiwan; i Department of Biochemical Science and Technology, National Chiayi University, Chiayi, 600355, Taiwan; j Graduate Institute of Natural Products, College of Pharmacy, Kaohsiung Medical University, Kaohsiung, 807378, Taiwan; k School of Chinese Medicine, National Yang Ming Chiao Tung University, Taipei, 112304, Taiwan; l Department of Pharmacy, School of Pharmaceutical Sciences, National Yang Ming Chiao Tung University, Taipei, 112304, Taiwan

**Keywords:** Coptidis Rhizoma, Cognitive impairment, Neurodegeneration, Sleep deprivation. glymphatic clearance, Hemoglobin

## Abstract

**Background:**

and Aim: Coptidis Rhizoma extract (CRE) is a traditional herbal medicine commonly used to treat insomnia and cognitive deficits. However, its protective effects and underlying molecular mechanisms in conditions such as sleep deprivation (SD) are not fully understood.

**Experimental procedure:**

This study intended to elucidate these mechanisms by combining functional assessments, imaging techniques, and genomic analyses in an animal model of sleep fragmentation-induced cognitive impairment. CRE's efficacy was compared with melatonin, an established neuroprotective agent.

**Results and conclusion:**

Our results demonstrated that SD significantly impaired cognitive functions and induced oxidative stress, inflammation, and multiple forms of neuronal cell death. Additionally, SD disrupted growth hormone (Gh) and hemoglobin (Hb) synthesis, impaired glymphatic clearance, and promoted accumulation of amyloid β-peptide (Aβ_1-42_), collectively contributing to neuronal inflammation and degeneration. Oral administration of CRE (0.5–1.5 g/kg/day) and intraperitoneal melatonin (10 mg/kg/day) for 15 days effectively reversed these adverse effects by restoring Gh and Hb synthesis, TH- and DCX-positive neurons, and glymphatic function, reducing inflammation and neurodegeneration, and normalizing genes and proteins involved in neurotransmission, oxygen transport, DNA repair, cellular metabolism, structural integrity, and neuronal function. However, neither treatment restored the downregulated expression of Gh-related genes, indicating limited effects on hypothalamic-pituitary regulation. In conclusion, these findings highlight the potential therapeutic role of CRE in alleviating cognitive and physiological impairments caused by sleep deprivation.

## Highlights

• CRE restores glymphatic clearance and neuronal homeostasis, reversing sleep deprivation–induced cognitive impairment.
• CRE enhances hemoglobin synthesis, oxygen transport, and cellular repair.
• CRE reduces oxidative stress, neuroinflammation, and amyloid β accumulation, preventing neuronal damage.
• CRE mitigates SD-related neural and metabolic dysfunctions, supporting cognitive and physiological recovery.

## Graphical abstract

**Figure:**
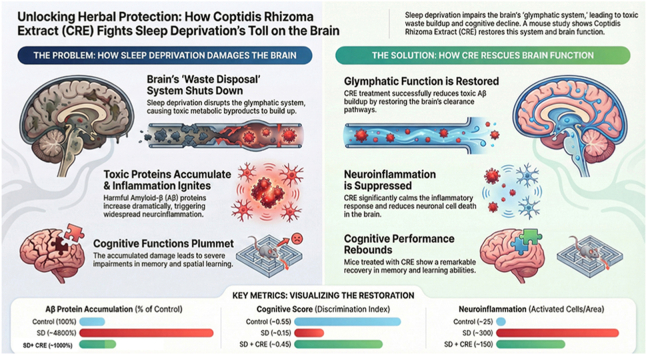

## 1 Introduction

Sleep is essential for cognitive health, particularly memory functions. Sleep deprivation (SD) has been linked to chronic conditions such as cardiometabolic dysfunction, mental health disorders, neurological diseases, and dementia.^1^, ^2^, ^3^, ^4^, ^5^, ^6^, ^7^ SD significantly impairs memory consolidation and hippocampal function, crucial for learning and memory, by disrupting neurotransmitter activity, protein synthesis, and neural adaptability.^8^ Key molecular disruptions include altered NMDA receptor activity, increased PDE4A enzyme levels, downregulated mTOR signaling, and heightened cellular stress responses.^9^^,^^10^ Additionally, SD-driven neuroinflammation exacerbates cognitive deficits by elevating inflammatory markers and oxidative stress.^11^^,^^12^ Despite these advances, the exact mechanisms involved are still not fully understood, and therapeutic options remain scarce. Melatonin is currently one of the few pharmacological treatments available to counteract memory impairment resulting from chronic sleep deprivation.^13^ It was therefore included as a reference drug in this study. Further research into these mechanisms is vital to developing effective therapeutic interventions.

Coptidis Rhizoma, a principal component of Huang-Lian-E-Jiao Decoction, has traditionally been used to treat insomnia and related neurological symptoms.^14^ Modern research confirms that this herb, partially due to its berberine content, improves sleep quality, reduces anxiety, and provides neuroprotective effects by decreasing oxidative stress and enhancing cognitive function.^15^ Given that SD induces cognitive impairment primarily through neuroinflammation, oxidative stress, and neuronal dysfunction, investigating the effects of Coptidis Rhizoma extract on SD-induced cognitive deficits is scientifically justified and promising.

This study examines whether Coptidis Rhizoma extract (CRE) can effectively alleviate cognitive deficits caused by SD in an animal model. Specifically, we investigate CRE's capacity to counteract key consequences of SD, including inflammation, neuronal damage, and oxidative stress, impaired waste clearance in the brain, amyloid-β (Aβ) accumulation, hormonal imbalance, disrupted neurotransmitter function, and reduced cellular repair processes. Additionally, we evaluate whether CRE provides superior therapeutic benefits compared to melatonin, a current standard treatment for SD-related cognitive impairment.

## 2 Materials and methods

### 2.1 Preparation of Coptidis Rhizoma extract (CRE)

Coptidis Rhizoma (the rhizome of *Coptis chinensis* Franch.) was purchased from the Dihua Street Market in Taipei, Taiwan. The sample was authenticated by Dr. Chia-Ching Liaw from the National Institute of Chinese Medicine (NRICM), MOHW, Taiwan, and a voucher specimen (No. NRICM-NHP-01086) was deposited at NRICM. Coptidis Rhizoma (100 g) was refluxed with 95% ethanol (450 mL) for 60 min. This process was repeated twice, and the combined extracts were concentrated under reduced pressure at 50°C to obtain the crude extract of Coptidis Rhizoma (CRE).

### 2.2 HPLC fingerprint profiles and major components quantification of CRE

**Fig. 1:**
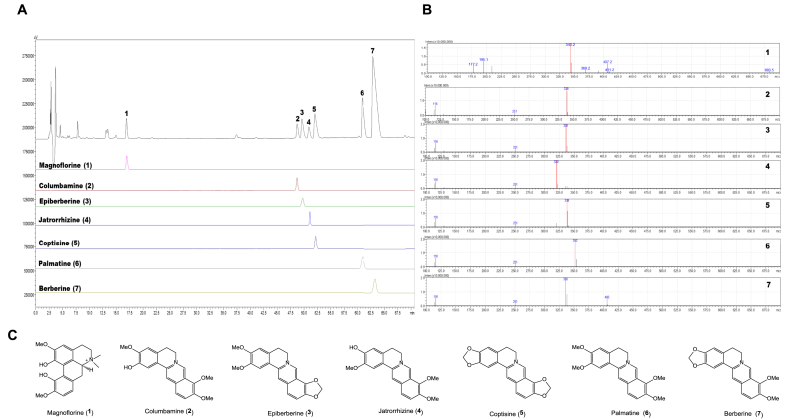
The HPLC fingerprint of Coptidis Rhizoma Extract (CRE) and mass spectra of compounds **1**–**7**. (A) The HPLC profile on 210 nm of UV wavelength with the reference compounds. (B) The mass spectra of seven major peaks. (C) The chemical structure of the seven major compounds, **1**: magnoflorine, **2**: columbamine, **3**: epiberberine, **4**: jatrorrhizine, **5**: coptisine, **6**: palmatine, and **7**: berberine.

To analyze the significant components of CRE, the HPLC profile of CRE (Fig. 1) was established by a Shimadzu Nexera-*i* liquid chromatograph (LC-2050C 3D, Shimadzu, Kyoto, Japan) equipped with a Chromanik Sunniest C_18_ column (ID 4.6 mm × 250 mm) at 30 °C. The mobile phase consisted of (A) distilled water with 0.1% TFA (trifluoroacetic acid) and (B) acetonitrile with 0.1% TFA, applied in a gradient: 0–30 min, 15–20% B; 30–50 min, 20–25% B; 50–60 min, 25–35% B; 60–70 min, 35% B. The sample concentration was 10 mg/mL, with a flow rate of 1.0 mL/min and an injection volume of 5 μL. Seven major compounds, including magnoflorine (**1**, 2.00%, CAS No.: 4277-43-4), columbamine (**2**, 1.91% CAS No.:3621-36-1), epiberberine (**3**, 3.22% CAS No.:6873-09-2), jatrorrhizine (**4**, 2.21% CAS No.:3621-38-3), coptisine (**5**, 4.54% CAS No.:6020-18-4), palmatine (**6**, 8.41% CAS No.:10605-02-4), and berberine (**7**, 26.42% CAS No.:633-65-8), were confirmed by the reference compounds and mass data using a Shimadzu Nexera-*i* LC-40D *xsi* system with an LCMS-8045 mass spectrometer (Table 1).

**Table 1:** Major components analysis of CRE.

Compound	Retention time	mg/g	Percentage (%)
Magnoflorine (**1**)	16.87 min	20.00 ± 0.1	2.00%
Columbamine (**2**)	48.51 min	19.1 ± 0.1	1.91%
Epiberberine (**3**)	49.79 min	32.2 ± 0.1	3.22%
Jatrorrhizine (**4**)	51.09 min	22.1 ± 0.1	2.21%
Coptisine (**5**)	52.39 min	45.4 ± 0.1	4.54%
Palmatine (**6**)	61.73 min	84.1 ± 0.1	8.41%
Berberine (**7**)	63.51 min	264.2 ± 0.1	26.42%

### 2.3 Animal grouping and induction of sleep deprivation (SD) in mouse model

**Fig. 2:**
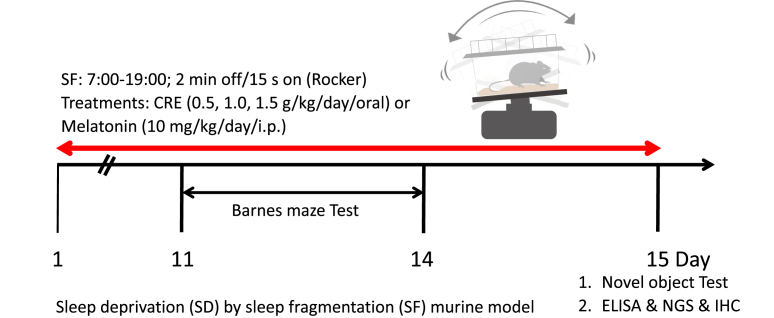
Effects of CRE on sleep deprivation-induced changes in mice. The experimental design involved ICR mice subjected to sleep fragmentation (SF: 7:00–19:00; 2 min off followed by 15 s on a rocker) to induce sleep deprivation (SD). Mice were divided into six groups: (1) saline control (CTX), (2) SD with saline treatment (SD + saline), (3–5) SD with oral CRE treatment at 0.5, 1.0, or 1.5 g/kg [SD + CRE (0.5 g, 1.0 g, or 1.5 g)], and (6) SD with intraperitoneal melatonin treatment (10.0 mg/kg/day, SD + Mela). CRE (0.5–1.5 g/kg) was administered orally once daily for 15 consecutive days, beginning on day 1. On day 15, mice were euthanized, and serum and brain tissue samples were collected for analysis following behavioral assessments. CRE: Coptidis Rhizoma extract (CRE); Mela: melatonin.

Male ICR mice (6–8 weeks old, 22–25 g) were obtained from the National Laboratory Animal Breeding and Research Center (Taipei, Taiwan) and acclimated for one week under controlled conditions (12-h light/dark cycle, 21–23°C, 60–70% humidity) with free access to standard chow (MFG, Oriental Yeast Co., Ltd, Japan) and water. The study was approved by the Animal Research Committee of the National Research Institute of Chinese Medicine (Approval No.: NRICM-IACUC-111-912-3). Animals were randomly assigned to experimental groups, and behavioral assessments were conducted by investigators blinded to the treatment conditions. Mice were randomly divided into six groups (n = 10 per group): (1) control (CTX), (2) sleep deprivation (SD) + saline, (3–5) SD + CRE (0.5, 1.0, or 1.5 g/kg/day for 15 days), and (6) SD + melatonin (ML, 10 mg/kg/day for 15 days).^16^^,^^17^ Sleep deprivation was induced via sleep fragmentation using a mechanized tilting platform (Stuart Scientific Platform Rocker STR6) controlled by a programmable cycle timer (Mini Asymmetrical Cycle Timer, AC/DC 12–240V GRT8-S2, Regun), operating on a 2 min OFF/15 s ON cycle from 7:00 to 19:00 throughout the entire experimental period.^18^ Body weight was recorded daily for 15 days; Barnes maze testing was performed on days 11 and 14, and novel object recognition on days 14 and 15. The experimental design is shown in Fig. 2.

### 2.4 Barnes maze test

The Barnes maze test was used to evaluate spatial learning and memory by measuring escape latency across different groups from days 11–14 following sleep deprivation (SD) or sham treatment (refer to Fig. 2 for the test schedule). The apparatus consisted of a circular platform (91.5 cm in diameter, 115 cm in height) with 20 evenly spaced holes along its perimeter (San Diego Instruments). To create an aversive environment, the maze was illuminated with bright overhead lighting (450 lux), and white noise (85 dB) was played. A designated escape chamber, positioned beneath one of the holes, was assigned to each mouse. Visual cues were placed on the surrounding walls and curtains to aid navigation. At the beginning of each trial, mice were confined within a white cylindrical container (10.5 cm in diameter) before being released into the maze. Behavioral performance was recorded and analyzed using the ANY-maze v 4.99 video-tracking system, and all experiments were conducted in a temperature-controlled room (20°C**)** designated for behavioral testing.^3^

### 2.5 Novel object recognition (NOR) test

The novel object recognition test was conducted following established protocols and consisted of three phases: training (memory acquisition) on day 14, a delay period (memory consolidation), and the test session (memory expression) on day 15.^19^^,^^20^ Prior to training, each mouse was individually acclimated to a behavioral observation box (40 × 60 × 40 cm) for 5 min, during which their movement was recorded by a camera. After habituation, mice were briefly returned to their home cages while the testing area was cleaned with 70% ethanol. During the training phase, each mouse was introduced to an arena containing two identical objects and allowed to explore them freely for 10 min. An animal was considered to be engaging with an object when its nose was within 2 cm of it. Mice who explored the objects for less than 10 s during the session were excluded from further testing. Following this phase, mice were returned to their home cages for the memory consolidation period. After 24 h, one of the previously presented objects was replaced with a novel object, and mice were reintroduced to the arena for 10 min. Their exploratory behavior was recorded and analyzed using the SMART v2.5.21 video-tracking system (Panlab, Spain). Recognition memory was assessed using the recognition index, calculated as (N or F)/(N + F) × 100%, where N and F represent the time spent exploring the novel and familiar objects, respectively. Typically, mice with intact recognition memory exhibit a preference for the novel object over the familiar one. Discrimination index was calculated according to the following equation: Discrimination index = (Time spent of exploring novel object – Time spent of exploring familiar object)/(Time spent of exploring novel object + Time spent of exploring familiar object).

### 2.6 Immunohistochemistry (IHC)

For immunohistochemical analysis, 15 to 20 serial brain sections (30 μm thick) were obtained from corresponding regions from the same parts of the brains near hippocampus (at the same rostrocaudal levels: bregma −1.5 to −1.9 mm) across all experimental groups. Tissue preparation followed a standardized protocol, including fixation, permeabilization, and blocking, to ensure optimal antibody binding. Selected sections were then randomly chosen for overnight incubation at 4 °C in phosphate-buffered saline (PBS) containing 3% albumin. After incubation, sections were stained with primary antibodies targeting specific protein markers to facilitate subsequent analysis including pGSK3 (Ser9) (1 : 100), Mki67 (1 : 100), SCN5a (1 : 100), and RPS2 (1 : 100), all obtained from Santa Cruz (Irvine, CA, USA); NLRP3 (1 : 200) from iREAL (Taipei, Taiwan); pNFκB P65 (1 : 100), obtained from BD (San Diego, CA, USA); GFAP (1 : 100) and PRMT8 (1 : 100), obtained from Cell Signaling Technology Inc. (MA, USA); Doublecortin (DCX, 1: 1000), IBA-1 (1 : 100), AQP4 (1 : 200), Aβ1-42 (1 : 100), pmTOR (1 : 100), and NeuN (1 : 200), obtained from Abcam (Cambridge, UK); IL-1β (1 : 200) from BioLegend (San Diego, CA, USA); active Caspase 3 (truncated, 1 : 100), obtained from Calbiochem (USA); p70S6K (1 : 100) from Millipore (USA); and Tyrosine hydroxylase (TH, 1 : 1000), obtained from MyBiosource (San Diego, CA, USA). Tissue sections were thoroughly washed and subsequently incubated with secondary antibodies conjugated with Alexa Fluor 488, 555, or 647 (Cell Signaling Technology, Danvers, MA, USA). After staining, the sections were mounted on coverslips using a medium containing 4’,6-diamidino-2-phenylindole (DAPI) to visualize cell nuclei. Imaging was performed using a Zeiss LSM780 confocal laser-scanning microscope (Carl Zeiss, Jena, Germany).

For quantitative analysis, Zen 2011 (Black Edition, Carl Zeiss MicroImaging, 1997–2011) and AlphaEase FC (Alpha Innotech, San Leandro, CA, USA) were used to identify, count, and determine the area occupied by immunopositive cells or the proportion of the total area displaying immunoreactivity. This analysis was conducted across the entire field of view in predefined regions of interest for each experimental group. Imaging was performed at 30–100 × magnification, with 3–5 independent replications for reliability. Each fluorescence channel was converted to green fluorescence using Zen 2011, Black Edition (Carl Zeiss) to quantify fluorescence-positive areas. AlphaEase FC software (version 4.0, Alpha Innotech) was then used to adjust contrast and sensitivity, defining the total analysis area. The software subsequently calculated the proportion of fluorescence-positive regions within the designated area.

### 2.7 Determination of hemoglobin (Hb) and growth hormone (Gh) level

Blood samples were collected under anesthesia on the final day before sacrifice and analyzed for growth hormone (Gh) and hemoglobin (Hb) levels using mouse-specific ELISA kits (MyBioSource, San Diego, CA, USA; Cat. Nos. MBS160945 and MBS107766, respectively). EDTA or heparin was used as an anticoagulant. For Hb measurement, samples underwent ultrasonication or freeze-thaw cycles to lyse cells, followed by centrifugation at 1000 *× g* for 20 min to isolate the supernatant. ELISA plates and reagents were equilibrated to room temperature (18–25°C) before assay. For Hb, 50 μL of standards or samples and 100 μL of HRP-conjugate reagent were added to designated wells, incubated at 37°C for 60 min, and washed four times. Chromogen solutions A and B (50 μL each) were sequentially added, incubated at 37°C for 15 min, and then stopped with 50 μL stop solution. Optical density (OD) was measured at 450 nm. For Gh, 50 μL standards, 40 μL samples, 10 μL anti-Gh antibody, and 50 μL streptavidin-HRP were added, incubated at 37°C for 60 min, and washed five times. Substrate solutions A and B were added (50 μL each), incubated for 10 min, and stopped. OD was read at 450 nm within 10 min.

### 2.8 RNA expression and next-generation sequencing (NGS)

For the NGS analysis, whole hippocampus tissues were collected from mice in all treatment groups. RNA was extracted from these samples, and its purity and concentration were assessed using a SimpliNano™ Biochrom Spectrophotometer (Biochrom, MA, USA). Subsequently, the prepared RNA samples from all experimental groups were subjected to next-generation sequencing (NGS). To assess RNA quality and quantity, a SimpliNano™ Biochrom Spectrophotometer (Biochrom, MA, USA) was used for sample purity evaluation across the four experimental groups. RNA integrity and degradation were further monitored using a Qsep 100 DNA/RNA Analyzer (BiOptic Inc., Taiwan). For sequencing library preparation, total RNA was processed with the KAPA mRNA HyperPrep Kit (KAPA Biosystems, Roche, Basel, Switzerland). High-throughput sequencing was conducted using the Illumina NovaSeq 6000 platform, generating raw sequence data. FastQC and MultiQC were utilized for quality control assessment, while Trimmomatic (v 0.38) was applied to filter and refine paired-end reads for further processing. Read alignment against the reference genome was executed using HISAT2 (v 2.1.0), followed by quantification of mapped reads for each gene using FeatureCounts (v 2.0.0). Differential gene expression analysis between case and control groups was performed with DEGseq (v 1.40.0) or DESeq2 (v 1.26.0).^21^

### 2.9 Statistical analysis

All data in this study are expressed as mean ± SEM. Group differences were assessed using a one-way analysis of variance (ANOVA), followed by the Newman–Keuls *post hoc* test for multiple comparisons. A *p*-value of less than 0.05 was considered statistically significant.

## 3 Results

### 3.1 Major component analysis and HPLC fingerprint profile of Coptidis Rhizoma extract (CRE)

The chemical composition of Coptidis Rhizoma Extract (CRE) was established by high-performance liquid chromatography (HPLC) with diode array detector and mass spectrometer (Fig. 1). The seven major peaks have been compared by the reference compounds, including one aporphine alkaloid, magnoflorine (**1**, R_t_: 16.87 min) and six berberine derivatives, columbamine (**2**, R_t_: 48.51 min), epiberberine (**3**, R_t_: 49.79 min), jatrorrhizine (**4**, R_t_: 51.09 min), coptisine (**5**, R_t_: 52.39 min), palmatine (**6**, R_t_: 61.73 min), and berberine (**7**, R_t_: 63.51 min). LC-MS analysis further confirmed these components through consistent mass ion peaks with *m/z* values of 342 (**1**), 338 (**2**), 336 (**3**), 320 (**4**), 338 (**5**), 352 (**6**), and 336 (**7**), respectively. Quantitative analysis showed berberine as the predominant berberine (26.42%), followed by palmatine (8.41%), coptisine (4.54%), epiberberine (3.22%), jatrorrhizine (2.21%), magnoflorine (2.00%), columbamine (1.91%) (Table 1).

### 3.2 Effect of CRE on SD-induced alterations in body weight

**Fig. 3:**
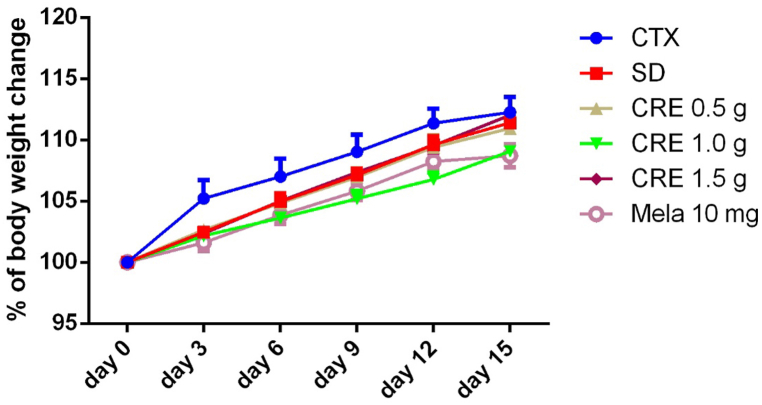
Effects of CRE on body weight changes over 15 days following SD induction in mice. The animal grouping follows the design outlined in Fig. 2. Body weight was measured daily across all groups for 15 days. Data are presented as mean ± S.E.M. (n = 10 per group). One-way ANOVA analysis showed no significant differences among groups (*p* > 0.05). CRE: Coptidis Rhizoma extract (CRE); Mela: melatonin.

Body weight changes are a key safety indicator in mice studies, reflecting overall health, treatment tolerability, and potential toxicity. Significant weight loss may indicate adverse effects, while stable weight suggests good treatment safety. To assess the safety of CRE in protecting against SD-induced cognitive dysfunction and its impact on hormone release, CRE at doses of 0.5–1.5 g/kg body weight, or the reference drug melatonin at a dose of 10.0 mg/kg body weight, was administered for fifteen consecutive days. SD caused a slight decrease in body weight across all groups compared to the control group. However, this difference was not statistically significant (*p* > 0.05) among these groups (Fig. 3).

### 3.3 Effect of CRE on SD-induced cognitive impairment

**Fig. 4:**
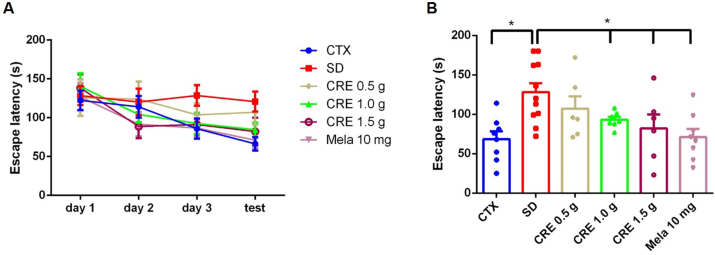
Effects of CRE on escape latency in the Barnes maze test over four trials following SD induction in mice. (A) The animal grouping follows the design outlined in Fig. 2. Escape latency (seconds) was measured on days 11 (day 1), 12 (day 2), 13 (day 3), and 14 (test) during the test phase over a 15-day period. Data are presented as mean ± S.E.M. (n = 10 per group). One-way ANOVA analysis showed no significant differences among groups (*p* > 0.05). (B) However, on day 14, a significant difference was observed among groups, as determined by one-way ANOVA followed by a *post hoc* Newman–Keuls test (∗, *p* < 0.05). CRE: Coptidis Rhizoma extract (CRE); Mela: melatonin.

**Fig. 5:**
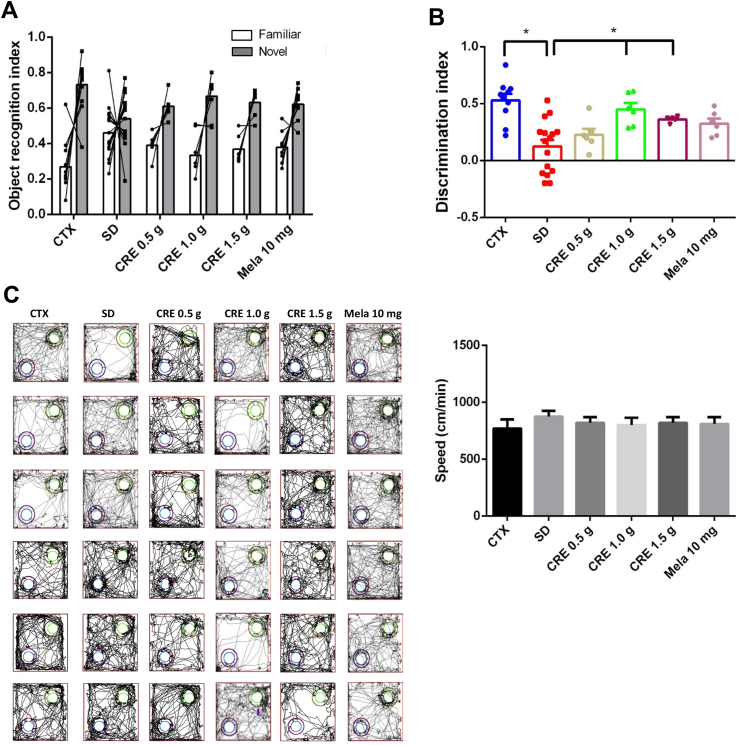
Effects of CRE in the novel object recognition (NOR) test over two trials following SD induction in mice. (A) The animal grouping follows the design outlined in Fig. 2. The Novel Object Recognition (NOR) test was conducted on days 14 and 15. On day 14, mice were exposed to two familiar objects, and on day 15, one familiar object and one novel object were presented. NOR index was described in the method. Data are shown as mean ± S.E.M. (n = 6–15 per group). (B) On day 15, a significant difference was observed among the groups, as determined by one-way ANOVA followed by a *post hoc* Newman–Keuls test (∗, *p* < 0.05). (C) Effects of CRE and melatonin on locomotor activity in sleep-deprived mice. Representative movement tracking plots (left) and average locomotion speed (right) for control (CTX), SD + saline, SD + CRE (0.5, 1.0, 1.5 g/kg), and SD + mela (10 mg/kg) groups. Locomotor speed (cm/min) was measured over 3 min in an open-field test. No significant differences were observed among groups (one-way ANOVA, *p* = 0.805). CRE: Coptidis Rhizoma extract (CRE); Mela: melatonin.

The Barnes maze test and novel object recognition (NOR) test were conducted to assess the effects of SD on cognitive function in mice. During the training phase (Day 1–Day 3), SD caused a slight increase in escape latency compared to the control group, but the difference was not statistically significant (Fig. 4A, *p* > 0.05). Treatment with CRE or melatonin reduced escape latency to levels similar to those of the control group (Fig. 4A). On the test day (Day 4), however, the SD group showed a significant increase in escape latency compared to the control group (Fig. 4B, *p* < 0.05). Treatment with CRE (at 1.0 and 1.5 g/kg) or melatonin effectively reversed this increase, significantly reducing escape latency (Fig. 4B, *p* < 0.05). In the NOR test, the SD group exhibited a significant reduction in the discrimination index (< 0.2) compared to the control group (> 0.5) (Fig. 5). Treatment with CRE (at 1.0 and 1.5 g/kg) but not melatonin successfully restored the discrimination index to levels comparable to those of the control group (Fig. 5B, *p* < 0.05). In the locomotor test, the result showed no significant differences (Fig. 5C).

### 3.4 Effect of CRE on SD-induced changes in Gh and Hb production

**Fig. 6:**
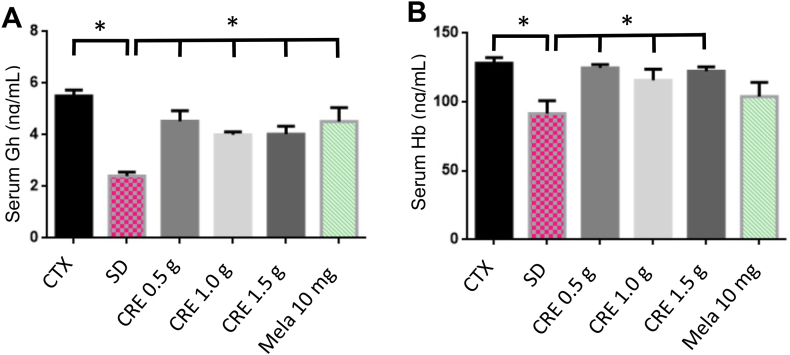
Effects of CRE on growth hormone (Gh) and hemoglobin (Hb) levels following SD induction in mice. Blood samples were collected from all groups on day 15 after the completion of tests. Gh (A) and Hb (B) concentrations were measured using ELISA kits as described in the methods section. The animal grouping: (1) saline control (CTX), (2) SD with saline treatment (SD), (3–5) SD with oral CRE treatment at 0.5, 1.0, or 1.5 g/kg [CRE (0.5 g, 1.0 g, or 1.5 g)], and (6) SD with intraperitoneal melatonin treatment (10.0 mg/kg/day, Mela 10 mg). Data are presented as mean ± S.E.M. (n = 6–10 per group). A significant difference was observed among groups, as determined by one-way ANOVA followed by a *post hoc* Newman–Keuls test (∗*p* < 0.05 as compared to SD group). CRE: Coptidis Rhizoma extract (CRE); Mela: melatonin.

To investigate the mechanisms behind CRE's protective effects against SD-induced cognitive impairment, we examined the expression of two key protective factors affected by SD: growth hormone (Gh) and hemoglobin (Hb).^22^^,^^23^ Our findings revealed that SD significantly reduced Gh and Hb levels (Fig. 6). However, CRE treatment effectively restored these levels to those observed in the control group. In contrast, melatonin had such an effect only on Gh level.

### 3.5 Effect of CRE on SD-induced changes in protective and detrimental protein expression

**Fig. 7:**
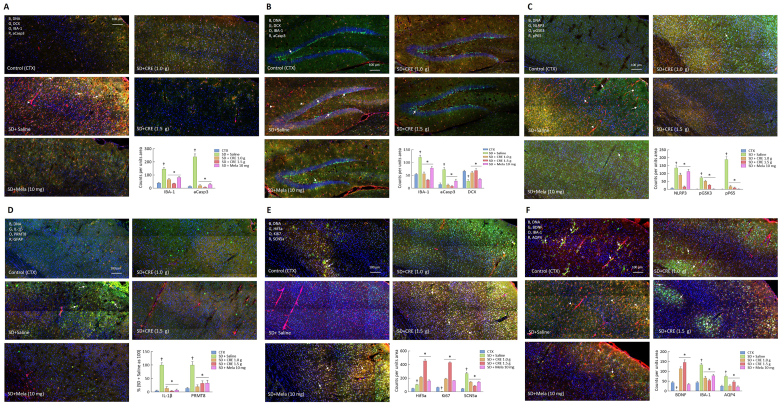

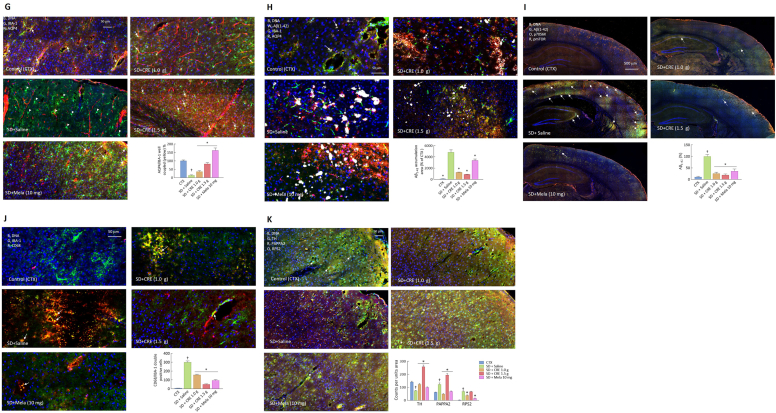
Effects of CRE on inflammation, oxidative stress, cell death, glymphatic dysfunction, and protective factors in mice following SD induction. The animal groupings follow the design outlined in Fig. 2. Representative confocal images of brain tissue illustrate changes in marker expression on day 15 post-SD induction: (A) Cortex and (B) hippocampus: expression of DCX (green), IBA-1 (orange), and active caspase-3 (aCasp3, red); arrowheads indicate aCasp3, while arrows mark IBA-1. The lower right panel provides a statistical summary. (C) Cortex: expression of NLRP3 (green), pGSK3 (orange), and pNF-κB P65 (red); arrowheads indicate pP65, and arrows indicate NLRP3. (D) Cortex: expression of IL-1β (green), PRMT8 (orange), and GFAP (red); arrows indicate IL-1β. (E) Cortex: expression of Hif3a (green), Ki67 (orange), and SCN5a (red); arrows indicate co-localization of Hif3a and Ki67. (F) Cortex: expression of BDNF (green), IBA-1 (orange), and AQP4 (red); arrowheads indicate IBA-1, while arrows indicate co-localization of BDNF and AQP4. (G) Cortex: expression of IBA-1 (green) and AQP4 (red); arrowheads indicate IBA-1, while arrows indicate co-localization of IBA-1 and AQP4. (H) Cortical deposition of amyloid-β_1-42_ (Aβ_1-42_; white (arrows indicated)), AQP4 (red), IBA-1 (green), and DNA (blue); the bar graph quantifies cortical Aβ_1-42_-positive area (expressed as % of Control). (I) Whole right brain section: expression of Aβ (green), and its associated signals p70S6K (orange), and pmTOR (red); arrows indicate co-localization of Aβ with p70S6K and pmTOR. The bar graph (right panel) quantifies Aβ-positive area as a percentage (%) of the SD + saline group. (J) Cortical regions stained for DNA (blue), IBA-1 (green), and CD68 (red), showing microglial activation patterns in different groups. SD markedly increased the number of IBA-1/CD68 double-positive activated microglia (yellow/orange; arrows), whereas CRE and melatonin treatment reduced microglial activation. The bar graph (right panel) quantifies IBA-1/CD68 double-positive cells per unit area. (K) Cortex: expression of TH (green), RPS2 (orange), and PAPPA2 (red); arrows indicate co-localization of TH and RPS2. The lower right panel provides statistical summaries for all markers. Data were analyzed using one-way ANOVA followed by a *post hoc* Newman-Keuls test (^†^, ∗*p* < 0.05 vs. corresponding sham or SD + saline group). CRE: Coptidis Rhizoma extract (CRE); Mela: melatonin.

To assess the impact of SD on oxidative stress, inflammation, and cell death pathways in the brain, we analyzed the cortex and hippocampal tissue samples from different groups (Fig. 7). SD resulted in significant microglial (IBA-1^+^) accumulation in the cortex (Fig. 7A) and hippocampus (Fig. 7B), along with increased apoptosis, as indicated by elevated active caspase-3 (aCasp3^+^) levels. Additionally, neuronal progenitor cell (DCX^+^) staining in SD + saline group was markedly reduced in the hippocampus (Fig. 7B). Treatment with CRE and melatonin provided dose-dependent benefits, reversing microglial accumulation, reducing apoptosis, and restoring neuronal progenitor cell (DCX^+^) numbers.

To further investigate the detrimental effects of SD, key markers of inflammation and cell death were analyzed. SD significantly upregulated inflammatory signals, including pNFκB P65 (pP65), pGSK-3, the pyroptosis marker NLRP3, and IL-1β. These increases were effectively mitigated by CRE and melatonin treatment (Fig. 7C & D). Additionally, SD markedly elevated PRMT8, a marker linked to oxidative stress-induced apoptosis and ferroptosis, as well as SCN5a, which may contribute to neuronal hyperexcitability under hypoxic stress. Both markers were significantly reduced with CRE and melatonin treatment (Fig. 7D & E), indicating that SD-induced oxidative stress and hyperexcitability were effectively mitigated by these interventions.

Sleep deprivation (SD) almost completely disrupted the colocalization of AQP4, a key marker of glymphatic function, with microglia (IBA-1), whereas this spatial association remained relatively intact in the sham group (Fig. 7F–H). The loss of AQP4–IBA-1 alignment suggests that SD may impair glymphatic integrity. This dysfunction was further supported by the widespread accumulation of amyloid-β (Aβ) and the upregulation of Aβ-related signaling markers, including pmTOR and p70S6K, across several brain regions (Fig. 7I), as well as the activation of microglia toward a more inflammatory phenotype, evidenced by increased IBA-1/CD68 double-positive cells (Fig. 7J). Treatment with CRE or melatonin partially restored glymphatic organization, reduced Aβ deposition and its downstream signaling activity, and attenuated microglial activation. Nevertheless, we acknowledge that Aβ accumulation is regulated by multiple biological pathways; therefore, although our findings are consistent with SD-induced glymphatic dysfunction, additional mechanisms may also contribute and warrant further investigation.

Finally, the impact of CRE and melatonin on protective factors was evaluated. SD significantly reduced the expression of Ki67 (Fig. 7E), BDNF (Fig. 7F), and tyrosine hydroxylase (TH) (Fig. 7K), a key enzyme in dopamine synthesis. Both treatments effectively restored their expression (Fig. 7E, F, & 7K). Conversely, SD increased the expression of PAPPA2 (Fig. 7K) (involved in IGF-mediated neuroprotection), SCN5a (Fig. 7E) (linked to neuronal excitability and hypoxia-induced hyperexcitability), Hif3a (Fig. 7E) (responses to oxygen deprivation, hypoxia), and RPS2 (Fig. 7K) (associated with hypoxia and oxidative stress response), suggesting these pathways were activated as compensatory mechanisms.

Melatonin markedly suppressed the SD-induced expression of PAPPA2, RPS2, and SCN5A, but further increased Hif3a. Similarly, CRE showed dose-dependent inhibition of SD-induced SCN5A, while further enhancing Hif3a expression. For PAPPA2, 1.0 g CRE significantly reduced its SD-induced upregulation, whereas 1.5 g CRE unexpectedly increased PAPPA2 relative to SD alone. For RPS2, 1.0 g CRE significantly decreased its SD-induced elevation, while 1.5 g CRE had no effect. These findings indicate that PAPPA2 and RPS2 are particularly sensitive to CRE treatment, but their differential, non-dose-dependent regulation indicates that further investigation is required. Together, these results suggest that CRE and melatonin modulated several SD-induced pathways, indicating their potential to counteract SD-evoked molecular stress responses.

### 3.6 Effect of CRE on SD-induced changes in gene expression

To better understand the systemic effects of CRE (1.5 g/kg) on SD-induced mice, we performed genome-wide transcriptome analysis using RNA-seq and next-generation sequencing (NGS) on the Illumina platform. This analysis aimed to analyze differentially expressed genes (DEGs) affected by SD and their potential modulation by CRE or melatonin treatment. NGS results showed that SD significantly upregulated two genes, *Apod* and *Abca8a*. CRE treatment fully reversed the upregulation of both, while melatonin only restored *Apod* to normal levels (Table 2). Conversely, SD markedly downregulated five genes: *Gh, Hbb, Hif3a, Hba,* and *Mki67*. CRE and melatonin treatment upregulated all except *Gh* and *Mki67* (Table 3). Additionally, CRE further enhanced the expression of nine genes potentially linked to protection against SD-induced cognitive impairment, including *Plin4, Th, Frmd7, Abi3, Prc1, Slc10a4, Cdkn1a, Alas2,* and *Ndst4* (Table 4).

**Table 2:** RNA-seq data indicating that sleep deprivation (SD) upregulates certain genes, while CRE and melatonin (Mela) partially reverse these effects.

Gene symbol	log2 Fold Change SD vs. sham (*p* adjust)	log2 Fold Change SD + CRE vs. SD (*p* adjust)	log2 Fold Change SD + Mela vs. SD (*p* adjust)
*Apod*	1.43 (4.26E-24)	−1.39 (1.40E-22)	−1.27 (1.01E-18)
*Abca8a*	1.25 (2.64E-05)	−1.17 (9.81E-05)	NA^a^

**Table 3:** RNA-seq data showing that sleep deprivation (SD) downregulates certain genes, while CRE and melatonin (Mela) partially restore their expression.

Gene symbol	log2 Fold Change SD vs sham (*p* adjust)	log2 Fold Change SD + CRE vs SD (*p* adjust)	log2 Fold Change SD + Mela vs SD (*p* adjust)
*Gh*	−7.14 (5.70E-03)	NA^a^	NA^a^
*Hbb*	−3.24 (9.11E-07)	3.18 (1.06E-06)	3.12 (2.13E-06)
*Hba*	−2.03 (3.98E-24)	2.22 (4.04E-29)	1.98 (1.42E-22)
*Hif3a*	−2.54 (4.95E-14)	2.86 (2.28E-18)	2.37 (5.83E-12)
*Mki67*	−1.54 (8.11E-03)	1.58 (3.32E-03)	NA^a^

**Table 4:** RNA-seq data showing that enhanced the expression of genes potentially linked to protection against SD-induced cognitive impairment, while CRE and melatonin (Mela) partially restore their expression.

Gene symbol	log2 Fold Change SD + CRE vs SD (*p* adjust)	log2 Fold Change SD + Mela vs SD (*p* adjust)
*Plin4*	3.28 (1.19E-07)	3.66 (3.98E-24)
*Th*	2.69 (1.65E-02)	NA^a^
*Frmd7*	2.62 (7.23E-03)	NA^a^
*Abi3*	2.51 (5.18E-03)	NA^a^
*Prc1*	2.01 (1.08E-02)	NA^a^
*Slc10a4*	1.77 (1.85E-03)	1.45 (1.60E-03)
*Cdkn1a*	1.63 (3.57E-05)	1.27 (5.17E-03)
*Alas2*	1.57 (8.70E-03)	NA^a^
*Ndst4*	1.51 (5.18E-10)	NA^a^

## 4 Discussion

The Huang-Lian-E-Jiao Decoction, originally documented in the Shang Han Lun, has a long-standing tradition of effectively addressing insomnia and restlessness associated with yin deficiency and internal heat. Its central ingredient, Coptidis Rhizoma, is particularly valued in traditional Japanese medicine for its calming effects and neuroprotective properties, partially attributed to the alkaloid berberine, known for mitigating cognitive deficits in neurodegenerative models.^24^ In the current study, we investigated the therapeutic effects and mechanisms of Coptidis Rhizoma extract (CRE) using an animal model of sleep deprivation (SD)-induced cognitive impairment. Consistent with previous literature, SD resulted in notable oxidative stress, neuroinflammation, disrupted glymphatic clearance, neuronal damage, and cognitive deficits. The observed cognitive deficits were confirmed to be independent of general motor performance, as no significant differences were detected in locomotor activity (Fig. 5C). Our findings suggest that CRE administration is associated with improvements in multiple pathological outcomes, including partial restoration of redox balance, enhancement of glymphatic system function, attenuation of inflammatory responses, potential reversal of dopaminergic damage in the brain, and ultimately, improvement in cognitive deficits. These observed benefits appeared to surpass those of melatonin, a well-recognized neuroprotective agent. Collectively, our results provide supportive scientific evidence correlating with the traditional use of Huang-Lian-E-Jiao Decoction, highlighting the translational promise of CRE in addressing cognitive impairments linked to sleep disorders.

The chemical composition of CRE was characterized using HPLC and LC-MS analyses, identifying seven isoquinoline alkaloids, including six berberine derivatives and one aporphine. These alkaloids, such as berberine, epiberberine, coptisine, and magnoflorine, are well-documented for their antioxidant, anti-inflammatory, and anti-apoptotic activities, properties which likely underpin CRE's observed therapeutic effects such as restoring oxidative balance and reducing neuroinflammation.^25^ Oral dosing of CRE at 0.5, 1.0, and 1.5 g/kg/day was selected based on pharmacological efficacy and established safety margins. Previous studies report an acute oral LD_50_ of approximately 2.95 g/kg in mice, indicating our dosing is safely below toxic levels.^26^ Furthermore, oral administration studies of berberine hydrochloride at around 200 mg/kg have shown substantial systemic exposure, supporting the pharmacological relevance of our chosen doses. Thus, the selected dose range is both safe and biologically meaningful, providing a sound basis for evaluating CRE's therapeutic potential *in vivo*.

SD downregulated Hb synthesis-related genes (*Hba*, *Hbb*), contributing to hypoxia and oxidative stress. This impairment may result from dysregulated erythropoietin (EPO) synthesis, disrupted hypoxia-inducible factor (Hif) regulation (*e.g.* Hif3a), increased oxidative stress, systemic inflammation, hormonal imbalances (e.g. melatonin deficiency), and altered iron metabolism.^27^

We found that the number of Hif3a-positive cells was significantly increased in the SD group compared with the control group (Fig. 7E), whereas mRNA analysis showed that Hif3a transcript levels were reduced in the SD group (Table 3). The differences between decreased mRNA expression and increased protein levels of Hif3a may be due to post-transcriptional regulation, transcriptional regulation, translation efficiency or protein degradation. The protein abundance of HIF family members is known to be tightly regulated by multiple factors, including oxygen availability and metabolites such as α-ketoglutarate, through post-translational modifications that critically control protein stability and degradation.^28^ However, the mechanisms underlying the divergent mRNA and protein expression of Hif3a remain to be elucidated. The increased Hif3a-positive staining suggests elevated Hif3a protein levels in the SD group relative to controls, and this increase was further enhanced by both CRE and melatonin treatment, implying that Hif3a protein may be involved in a compensatory regulatory mechanism in response to sleep deprivation. These results suggest that CRE and melatonin may enhance Hb expression by modulating these pathways. Both treatments restored *Hba* and *Hbb* gene expression, improving oxygen transport and antioxidant defenses while regulating Hif3a to support metabolic recovery.^29^

To the best of our knowledge, this is the first report showing the disruption of the glymphatic system in SD mice, evidenced by the spatial separation of IBA-1 (a microglial marker) and AQP4 (an astrocytic and glymphatic marker), underscores the detrimental impact of sleep deprivation on cerebrospinal fluid (CSF) exchange and metabolic waste clearance.^30^^,^^31^ Furthermore, the observed colocalization of the microglial activation marker CD68 with IBA-1 in affected brain regions (Fig. 7J) strengthens the interpretation of microglial involvement and provides preliminary functional insight into their contribution to the neuroinflammatory response.

Melatonin has been shown to enhance Aβ clearance via glymphatic-lymphatic drainage.^32^ Under normal conditions, resting microglia actively survey the brain parenchyma, interacting with astrocytes and neurons.^33^ This coordinated activity is essential for glymphatic function. Our study is the first to demonstrate that CRE and melatonin partially restore microglial-astrocyte interactions, enhance glymphatic clearance, and reduce Aβ accumulation, further supporting their neuroprotective effects.

SD also triggers the upregulation of protective genes to counteract neural stress. Among these, ApoD and ABCA8A help mitigate oxidative stress and regulate lipid homeostasis.^34^^,^^35^ However, excessive expression can exacerbate neural damage and cognitive decline. CRE and melatonin prevent this by downregulating their expression, thereby reducing SD-induced impairments. Similarly, the upregulation of SCN5a (Nav1.5) and PAPPA2 reflects an adaptive response to SD. SCN5a, expressed in astrocytes, supports ionic homeostasis and protects against excitotoxicity, though its prolonged activation may intensify oxidative damage, which CRE mitigates by reducing astrocytic hyperactivity.^36^ PAPPA2, a key regulator of IGF signaling, likely compensates for SD-induced Gh decline.^37^^,^^38^ By modulating these responses, CRE helps maintain balance, preventing excessive compensatory mechanisms from exacerbating neural damage.

CRE and melatonin also regulate lipid metabolism by upregulating Plin4, stabilizing lipid storage, and reducing lipid peroxidation, thereby preserving neuronal integrity and synaptic function.^39^ CRE demonstrated broader therapeutic effects than melatonin did, selectively enhancing genes involved in neurotransmitter regulation (*Th*, *Slc10a4*), cellular repair (*Abi3*, *Prc1*, *Cdkn1a*), hemoglobin synthesis (*Alas2*), tissue stability (*Ndst4*), and neuronal development (*Frmd7*).^40^, ^41^, ^42^, ^43^, ^44^, ^45^, ^46^ These findings highlight CREs comprehensive biological impact, targeting multiple pathways involved in cognitive function, cellular resilience, and systemic stability. This makes it a stronger candidate than melatonin for mitigating SD-induced dysfunctions.

Despite these benefits, neither CRE nor melatonin restored Gh gene expression, which is critical for growth, metabolism, and neural repair. This observation suggests that their mechanisms may not fully engage the hypothalamic-pituitary axis (HPA), or that alternative therapeutic strategies may be necessary to address profound Gh dysregulation. Additionally, the use of bulk RNA-seq from whole-brain tissue limits the resolution of cell-type-specific responses, thereby constraining our ability to pinpoint the precise cellular pathways modulated by CRE. Future investigations utilizing pathway-specific pharmacological interventions or genetic manipulation approaches are warranted to elucidate causal mechanisms and refine our mechanistic understanding.

## 5 Conclusion

CRE effectively mitigates SD-induced neural and systemic dysfunctions by enhancing hormone secretion, glymphatic clearance, gene expression, metabolic recovery, and cellular repair. These findings underscore its potential as a therapeutic intervention for cognitive and physiological impairments caused by SD while emphasizing the need for further research to optimize its effects, particularly in regulating Gh-related pathways.

## CRediT authorship contribution statement

**Chih-Yuan Yang:** Investigation, Formal analysis, Data curation, Writing – original draft. **Yea-Hwey Wang:** Investigation, Formal analysis, Data curation, Validation, Supervision. **Kuo-Tong Liou:** Writing – original draft, Validation. **Shuo-En Hsu:** Methodology. **Cher-Chia Chang:** Investigation, Formal analysis, Data curation, Validation. **Yen-Yu Chen:** Investigation, Formal analysis, Data curation. **Terry B.J. Kuo:** Investigation, Formal analysis, Data curation. **Hung-Tse Huang:** sample preparation, Investigation, Formal analysis. **I-Wen Lo:** Investigation, Formal analysis. **Chia-Ching Liaw:** Writing – original draft, Writing – review & editing, Methodology, sample preparation, Investigation, Formal analysis. **Yuh-Chiang Shen:** Writing – original draft, Writing – review & editing, Methodology, Validation, Conceptualization, Supervision, Project administration. **Yi-Chang Su:** Resources, Supervision, Project administration.

## Funding

This research was supported by National Research Institute of Chinese Medicine, the Ministry of health and Welfare, Taiwan MOHW113-NRICM-M-325-123400, MOHW113-NRICM-M-315-000004, MOHW112-NRICM-M-315-000004).

## Declaration of competing interest

The authors declare no conflict of interest. The funders had no role in the study design, data collection and analysis, the decision to publish, or the preparation of the manuscript.

## Figures and Tables

**Table:** 

**Aβ**	Amyloid β	**AQP-4**	Aquaporin
**aCasp3^+^**	Active caspase-3	**BDNF**	Brain-derived neurotrophic factor
**CRE**	Coptidis Rhizoma extract	**CSF**	Cerebrospinal fluid
**DCX^+^**	Neuronal progenitor cell	**EDTA**	Diaminoethane-tetraacetic acid
**DEGs**	Differentially expressed genes	**Gh**	Growth hormone
**EPO**	Erythropoietin	**Hif3a**	Hypoxia-inducible factor 3 subunit alpha
**Hif**	Hypoxia-inducible factor	**Hb**	Hemoglobin
**HPA**	Hypothalamic–pituitary axis	**LTP**	Long-term potentiation
**HPLC**	High-performance liquid chromatography	**mTOR**	Mammalian target of rapamycin
**NGS**	Next-generation sequencing	**NOR**	Novel object recognition
***NLRP3***	NOD-like receptor protein 3	**OD**	Optical density
**NMDA**	*N*-methyl-D-aspartate	**RPS2**	Ribosomal protein S2
**PRMT8**	Protein arginine methyltransferase 8	***SCN5a***	Sodium channel protein type 5 subunit alpha
**SD**	Sleep deprivation	**TH**	Tyrosine hydroxylase
**TFA**	Trifluoroacetic acid	**UPR**	Unfolded protein response

## Data Availability

Data will be made available on request.
